# ‘At first I was overwhelmed, but then—I have to say—I did almost enjoy it’. Psychological needs satisfaction and vitality of student teachers during the first Covid-19 lockdown

**DOI:** 10.1007/s11218-021-09667-2

**Published:** 2021-10-20

**Authors:** Matteo Carmignola, Daniela Martinek, Gerda Hagenauer

**Affiliations:** 1 School of Education, University of Salzburg, Erzabt-Klotz-Str.1; A – 5020, Salzburg, Austria; 2 University College of Teacher Education, Akademiestr. 23-25; A – 5020, Salzburg, Austria

**Keywords:** COVID-19 lockdown, Distance learning, Basic psychological needs, Vitality, Student teachers, Teacher education

## Abstract

According to Self-Determination Theory, the satisfaction of the three basic psychological needs (BPN) is crucial for optimal personal and academic development, as well as well-being, which is associated with the perceived vitality. These dimensions can be fostered or hindered by contextual factors within the work, study and personal environment. During the first COVID-19 lockdown, most social contexts for student teachers were substantially altered, for which we hypothesised negative outcomes with regards to perceived basic needs satisfaction and vitality. To investigate changes in needs satisfaction and subjective vitality during distance learning, this research applied an explanatory sequential mixed-methods design combining a quantitative and qualitative study. For the quantitative study, two surveys were conducted; the first before the lockdown (*N* = 161) and the second after the lockdown (*N* = 146). The purpose of these surveys was to test the interrelations between need satisfaction, frustration and vitality before and during the distance learning by implementing a SEM-based mediation analysis. In addition, we elaborated on significant and unexpected findings of the quantitative study by interviewing 14 other student teachers, allowing for an in-depth and contextualised understanding of the psychological changes in and the triggering factors of the ‘corona-lockdown’. Results stress the relevance of physical activity and time spent outdoors for the perception of vitality which was supported by a more flexible time structure during distance learning. For the context of distance learning, this research emphasises the relevance of BPN also in the context of online-based learning where higher levels of interaction with fellow students and lecturers, a clear course structure and formative feedback proved to be essential for motivational and achievement-related outcomes.

## Introduction

The outbreak of the COVID-19 pandemic in the early months of 2020 resulted in a worldwide crisis across economic, political and social dimensions. In some countries, schools and universities were among the first public institutions to adopt lockdown measures by shifting all educational activities to web-based and distance learning formats (Murphy, 2020). In Austria, as in many other countries, this radical change was implemented within days (Pichl, 2020) and with (almost) no preparation and support. While the impact of the lockdown measures on academic outcomes can only be determined in the next years, many researchers postulate possible negative effects of these extraordinary circumstances on the well-being and mental health of school pupils and students in Higher Education (HE) (Fawaz et al., 2021; Kumar & Nayar, 2020).

In this research, we focused on a particular group of students in HE, namely student teachers (students who are majoring in teacher education). Ensuring the quality of teacher education is a global concern; thus maintaining high-quality instruction is not only crucial throughout initial teacher education but also during the current pandemic. More concretely, we aimed to investigate the impact of the ‘corona lockdown’ on student teachers’ perceived vitality and the satisfaction of their basic psychological needs (BPN). Challenging situations, such as studying in distance mode, can alter previously familiar ways for satisfying the needs for autonomy, competence and relatedness, which makes it even more important to consider the importance of psychological need satisfaction as a key factor for subjective well-being during times of change (Martela & Sheldon, 2019).

In doing so, we applied an explanatory sequential mixed-methods design (Creswell & Plano-Clark, 2018) to gain a comprehensive understanding of the changes linked with the lockdown from the student teachers' perspective. We expect that the results will not only be valuable to understand the extraordinary situation of distance learning during the pandemic, but will also provide important insights on how to design learning opportunities in HE with a distance modality that best fulfils student teachers’ BPNs.

## Vitality and basic psychological needs of student teachers

Ryan and Deci’s (2017) self-determination theory (SDT) is an organismic dialectical approach to motivation, personality development and health. Accordingly, it postulates that all individuals have an inner tendency to develop themselves through inner organisation and integration of the self with others (Ryan & Deci, 2000). Social contextual factors play an essential role in stimulating or hindering these processes (Ryan et al., 2010) and, for student teachers, these contextual factors concerning their study environment changed drastically during the COVID-19 lockdown in Austria (Pichl, 2020).

Contextual factors can be conducive to human development, as they contribute to the satisfaction of the three BPNs of autonomy, competence and relatedness (Ryan & Deci, 2011; Van den Broeck et al., 2016). The need for autonomy refers to perceiving oneself as the origin of one’s behaviour or being able to identify with requested behaviour and the connected aims and values. The need for competence is satisfied, when the interaction with social environments is experienced as effective and provides situations to explore one’s capabilities and the need for relatedness refers to the desire to be looked after by significant others or to be a carer or friend for others (Deci & Ryan, 2008).The *need for autonomy* is met when student teachers can follow their interests and integrated values and when they experience themselves as the originator of their behaviour (Deci & Ryan, 2008). Within SDT, autonomy (also called self-determination) does not equal freedom or independence: Student teachers can also experience autonomy if the impulse to act originates from someone else or is driven by external factors or forces, but only if they successfully integrate the requested behaviour with their self and can identify with the aims (Chirkov et al., 2003). If, on the other hand, self-determination is hindered by heteronomy and student teachers feel pressured or forced to meet externally imposed demands that they cannot identify with, this is detrimental for motivational self-regulation and personality development (Martinek et al., 2020). The student teachers’ need for autonomy is acknowledged if they receive meaningful choices and opportunities to work in a self-determined way and if they can pursue their interests in teaching and studying (Reeve, 2018).

The *need for competence* refers to feeling effective during the interaction with social environments and successfully experiencing a situation in which student teachers can explore their skills and capabilities (Ryan & Deci, 2000). Student teachers feel competent if they experience an individual sense of confidence and effectiveness in action, making them believe that they can successfully manage, for example, study-related tasks and assignments and challenges in practical training. Influential factors conducive for satisfying the need for competence are optimal challenges, manifold sources for constructive feedback, a clear structure with transparent aims, well-sequenced input and various support options, including guidance and encouragement (Cheon et al., 2020).

The *need for relatedness* is expressed through or by the desire to care for somebody and to be cared for by others (Deci & Ryan, 2008; Reeve, 2018). Student teachers can experience relatedness to colleagues (Klassen et al., 2012), lecturers and/or pupils during their practical training (Aldrup et al., 2017). Student teachers feel related to their peers in the context of the university if they experience acknowledgement and attention and enjoy the company of others with whom they are willing to share personal resources. In short, relatedness contributes to developing a sense of belonging and feelings of security (Hagenauer & Raufelder, 2021).

The *SDT* states that these three BPNs are innate and universal and their satisfaction significantly contributes to organismic growth and psychological well-being in terms of full functioning (Deci & Ryan, 2011; Van den Broeck et al., 2008; Vansteenkiste et al., 2020). Furthermore, psychological needs lead to proactive behaviour: Highly satisfied student teachers actively seek out study environments and challenges that provide (further) opportunities to experience autonomy, competence and relatedness. Although the approaches and methods to satisfy one’s needs differ from person to person, all student teachers profit from need-supportive study environments.

In addition to the concept of need *satisfaction*, the dimension of need *frustration* is crucial for the understanding of motivational and emotional processes (Cheon et al., 2019). When study environments are indifferent to need satisfaction, this leads to need dissatisfaction (Wang et al., 2019), whereas if social contexts actively contribute to a negation of BPNs, it results in need frustration. In this case, experienced need-thwarting behaviour is associated with maladaptive functioning, negative emotions and stress (Bartholomew et al., 2011; Haerens et al., 2015). Low or no need frustration and adequate satisfaction of the needs for autonomy, competence and relatedness are, according to SDT, essential for ideal development, mental health and physiological and psychological well-being (Vansteenkiste & Ryan, 2013). This is particularly true in times of uncertainty. Recent results of intervention studies introduced by Cantarero et al. (2021) and Behzadnia & FatahModares (2020) during the COVID-19 outbreak confirmed that psychological need satisfaction was positively associated with students’ well-being. Furthermore, increasing students’ need satisfaction led to an increase in students’ mental well-being, which corresponds with findings from Vermote et al. (2021).

The research in hand focuses specifically on one facet of psychological well-being – subjective *vitality* (Ryan & Deci, 2008). Vitality is classically defined as having mental and physical energy, including the experience of enthusiasm and a thirst for life (Ryan & Frederick, 1997). From an SDT perspective, psychological needs satisfaction and low or no need frustration are necessary nutriments for subjective vitality (Ryan et al., 2006, 2010). Student teachers experience psychological well-being in study environments that foster need satisfaction, which is seen within SDT as eudaimonic well-being or being in sync with one’s inner self to function effectively and lead a successful life (Ryan & Deci, 2001). Such psychological well-being is expressed by a feeling of vitality and psychological flexibility and is fostered by autonomous (learning) activities that contribute to need satisfaction.

Ryan and Deci (2008) described vitality as the energy available to the self and as a salient and functionally significant indicator of health and motivation. Subjective vitality is associated with positive affect (Watson & Tellegen, 1985), greater stimulation and productivity, better stress management and mental health and greater resilience to physical and viral stressors (Benyamini et al., 2000; Cohen et al., 2006; Penninx et al., 2000; Polk et al., 2005; Weinstein & Ryan, 2010). Vitality has diurnal cycles and is influenced by somatic factors, such as exercise, sleep patterns and diet, and by psychological factors, such as student teachers’ energy for controlling their behaviours and suppressing impulses (Ryan & Deci, 2008).

### The present research

During the COVID-19 lockdown, social contexts for student teachers offering physical and psychological resources were substantially altered. Although this provided student teachers with new opportunities to adjust to their preferred diurnal patterns, it also meant they needed to cope with serious restrictions concerning individual freedom, personal contacts and mobility. Likewise, familiar study environments changed drastically within a few days: Student teachers, lecturers and practitioners had to rearrange their courses and the general framework for studying amid high uncertainty. How these changes were experienced by the student teachers and how they affected the fulfilment of their BPNs and, in consequence, their vitality were at the core of the present research. Based on an explanatory sequential mixed-methods design (QUAN → QUAL; Creswell & Plano-Clark, 2018), we aimed to investigate the following main research questions:

How do student teachers’ experience the fulfilment and thwarting of their psychological needs before and during the lockdown? How is this related to their vitality as an indicator of subjective well-being?

#### Quantitative phase (Study 1): research questions and hypotheses

In the quantitative phase, we investigated whether study modes (face-to-face vs. distance learning) and living contexts (normal vs. lockdown measures) affected satisfaction and frustration of BPNs and, in consequence, perceived vitality.

**Fig. 1:**
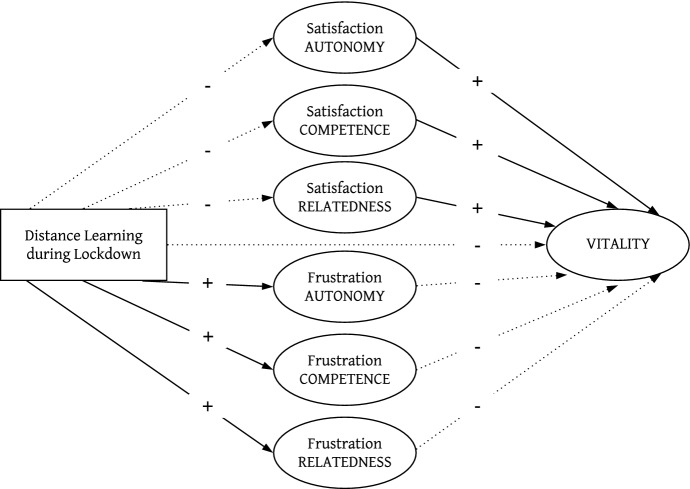
Conceptual model with expected regression paths for the predictor (distance learning during lockdown) and mediators (BPNSF) on perceived vitality

In detail, we tested the following hypotheses (see Fig. 1):

##### **Hypothesis 1**

Student teachers experience reduced satisfaction and increased frustration of BPNs during distance learning in the context of university studies (Wang et al., 2019).

##### Hypothesis 2

Student teachers experience decreased levels of vitality during distance learning (Ryan & Deci, 2008).

##### Hypothesis 3

Basic psychological needs will mediate the effect of distance learning on vitality by either buffering (needs satisfaction) or intensifying (needs frustration) the negative impact of distance learning and lockdown measures (Aldrup et al., 2017; Costa et al., 2015; Martela & Sheldon, 2019).

#### Qualitative phase (Study 2): research questions

In the qualitative phase, we aimed to obtain a contextualised understanding of the psychological changes and triggering factors during the corona lockdown. To this end, we investigated the following research questions that emerged from the analyses of the quantitative findings (outlined in detail in Sect. 4):

1. How do student teachers describe their vitality during the lockdown and what do they report as perceived supporting and hindering factors?
2. What changes did student teachers experience after lockdown measures were enacted?
3. How do student teachers perceive the fulfilment of their BPNs during the lockdown, with a particular focus on the need for competence?

## Method

### Mixed-methods design

The quantitative and qualitative approaches were combined in an explanatory sequential mixed-methods design (QUAN → QUAL; Creswell & Plano-Clark, 2018). In the first phase (QUAN), survey data were collected to test the interrelations between BPNs satisfaction and frustration and vitality. Furthermore, possible changes in these factors were examined by comparing the time before and after the corona lockdown (see Sect. 2.1.1). To this end, two measurements (t1 during the first two weeks of the summer semester [no pandemic restrictions]; t2 four weeks later, in the middle of the most restricted corona lockdown and distance learning mode) were compared to determine differences in BPNs satisfaction and frustration, and vitality. In the second phase (QUAL), end-of-semester interviews were conducted to better understand students’ experiences during the pandemic and how they may differ from the experiences during non-pandemic times (see Sect. 2.1.2).

**Fig. 2:**
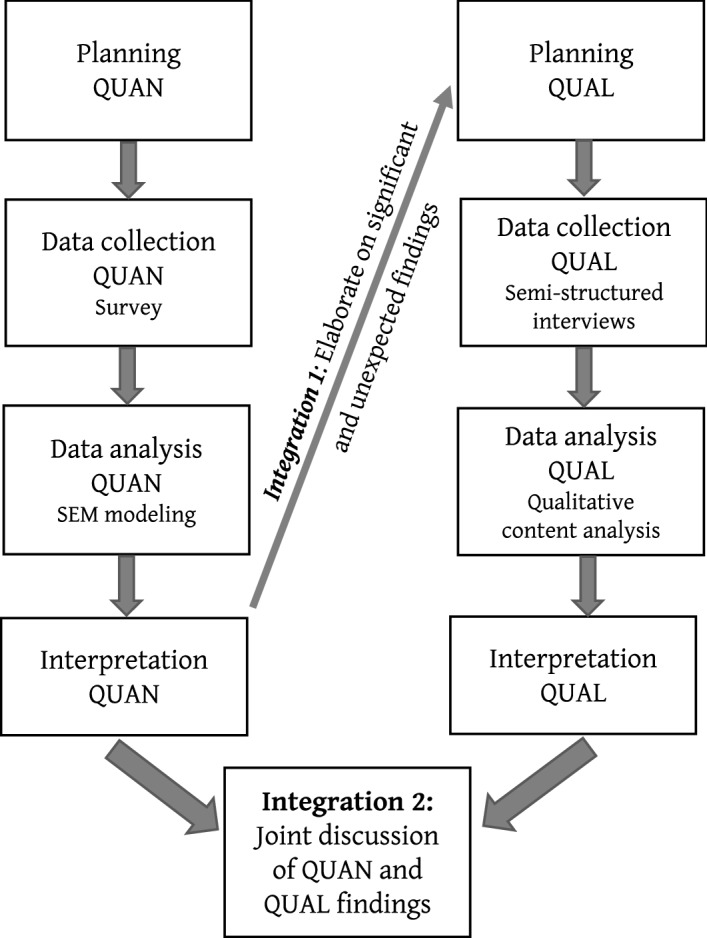
Explanatory sequential mixed-methods design of the present study

Integration is an inherent quality element of mixed-method studies (Fetters et al., 2013), which occurred twice in this study. First, integration took place after the analysis and interpretation of the data from the quantitative phase. The quantitative findings formed the basis for the derivation of research questions of the qualitative study and the development of the interview guide (see "Appendix 1"). Significant and unexpected findings from the quantitative strand were identified and explored during the qualitative strand of the research. Although this is a main rationale for the application of an explanatory sequential mixed-methods design (e.g. Creswell & Plano-Clark, 2018), it is seldom applied in research compared with other rationales (Bryman, 2007). A reason for this finding might be traced to the requested openness in the research design: Specific research questions of the second strand cannot be fixed in the planning phase of the study; they can only be specified after the completion of the first strand once the unexpected findings have been illuminated. Second, integration took place in the joint discussion of the findings resulting in meta-inferences to provide more complete answers on the research questions. In doing so, the complementary functions of both approaches could be combined fruitfully. The quantitative results led to generalisable findings, whereas the qualitative results allowed for an in-depth and contextualised understanding of the psychological changes in and the triggering factors during the corona lockdown. The design of the study is depicted in Fig. 2. More details on both the quantitative and qualitative strands of the study are provided in Sects. 3.2 and 3.3, respectively.

### Method of the quantitative phase (Study 1)

#### Participants of the quantitative phase (Study 1)

Two surveys were conducted in the summer term of 2020 with the same cohort of student teachers enrolled in a secondary school teacher university programme at an Austrian university. The first survey was conducted among regular studying conditions at the beginning of the summer term (first two weeks of March 2020) using an online-based questionnaire with a sample of 161 student teachers (69.9% female) with an average age of 24.8 years (*SD* = 2.28). Four weeks later (first two weeks of April 2020), the same cohort of student teachers were contacted again, which resulted in a sample of 146 student teachers (72.9% female). They participated in an online-based survey during the phase of distance learning as a result of the lockdown policies. Both samples were derived from the same cohort of student teachers, focusing on student teachers at the end of their undergraduate and beginning of their graduate studies. This specific sample was chosen as these student teachers have already progressed in their studies and thus, transition effects were not likely to occur as possible interfering variable affecting student teachers’ psychological need satisfaction and vitality. In addition, this cohort of student teachers have already acquired a substantial amount of (own) teaching practice during school practica, which is also an important factor that influences students’ need satisfaction and thwart in their studies as well as their vitality (Aldrup et al., 2017). In purposively selecting this specific cohort of student teachers in teacher education, significant interfering variables evoked by specific study conditions were hold constant.

The average age of this second sample (*M* = 23.4 years, *SD* = 3.43) differed slightly from the first; therefore, age was included as a covariate to control for possible sample bias in the students’ population. All participants completed informed consent forms and agreed to anonymous data processing for scientific purposes. As the study participation guaranteed anonymity, it was not possible to match the two datasets.

#### Measures

##### Vitality in the context of learning

The German version of the Subjective Vitality Scale (Ryan & Frederick, 1997) was used to measure student teachers’ vitality. The prompt specified the context by referring to attendance at university for the first sample and *distance learning* for the second sample. Participants answered seven items (e.g. *At this moment, I feel alive and vital.*) on a 7-point Likert-type scale (1 = *not true* to 7 = *very true*). For both samples, the instrument achieved high levels of scale reliability (ω_t1_ = 0.93; ω_t2_ = 0.93) and, as result of a CFA, a good factor validity (χ^2^(14) = 97.8; TLI = 0.92; CFI = 0.95; RMSEA = 0.14; SRMR = 0.04).

##### Basic psychological needs satisfaction and frustration (BPNSF)

Further, the German Basic Psychological Need Satisfaction and Frustration Scale (Chen et al., 2015; Heissel et al., 2018) was used to assess satisfaction and frustration for the needs of autonomy, competence and relatedness with regard to university studies, which—for the second measurement—was slightly modified to match the distance mode (prompt: “The following section covers your current experience in your [online] studies”). A total of 24 items were rated on a 5-point Likert scale (1 = *does not apply at all* to 5 = *applies completely*). Sample items and reliability coefficients are shown in Table 1.

**Table 1:** Sample items and scale reliabilities of the BPNSF

Subscales	Scale Reliability *ω*_t1_/*ω*_t2_	Sample Item
*Satisfaction*		
Autonomy	.76/.76	I feel that my decisions regarding my studies reflect what I really want.
Competence	.89/.87	I feel I can successfully complete difficult tasks for my study program.
Relatedness	.84/.81	I feel close and connected with other people from university who are important to me.
*Frustration*		
Autonomy	.85/.89	In my studies, I feel pressured to do too many things.
Competence	.76/.82	At university, I feel like a failure because of the mistakes I make.
Relatedness	.73/.73	I feel that people from university who are important to me are cold and distant towards me.

A model comparison based on CFA (Brown, 2006) indicated a significantly better fit (χ^2^(237) = 483; TLI = 0.91; CFI = 0.92; RMSEA = 0.06; SRMR = 0.06) for the six-factor solution measuring all six dimensions separately in comparison to a two-factor model with two aggregated factors for satisfaction and frustration. Furthermore, the authors opted against a second-order model to allow for estimation of the mediating effect of each need independently.

#### Study procedure and analysis

In the quantitative study, our main goal was to analyse the relationship between satisfaction and frustration of basic psychological needs (*BPNSFS)* towards the subjective vitality in the context of the university. We also wanted to determine whether distinct learning environments (i.e. face-to-face setting vs. distance learning due to the pandemic policies) were linked to different levels of vitality and needs satisfaction and frustration. To determine the effect of the independent variable (dichotomous: distance learning vs. face-to-face) and mediating variables (needs satisfaction and frustration), we implemented a mediation analysis based on structural equation models (SEMs). Additionally, the models included age as a covariate to control for possible sample bias (see Sect. 3.2.1).

Beforehand, the scales were examined with a confirmatory factor analysis (CFA): Brown (2006) as well as Flora and Flake (2017) recommend CFA to determine the construct validity of an instrument when the postulated structure of the latent measurement model is supported by theoretical hypotheses or prior validation studies, as was the case for the measures implemented in this research project. Furthermore, the authors opted for a SEM-based approached, as it has been demonstrated that SEM performs best in the analysis of mediation effects (Iacobucci et al., 2007). Moreover, SEM allows for the estimation of latent constructs and considers measurement errors; for this, an SEM-based approach is preferred to path analysis (Schreiber et al., 2006).

Besides chi-square statistics, we controlled model validity by following fit measures (i.e. TLI, CFI, RMSEA and SRMR [Brown, 2006]). Mediation effects were interpreted according to the typology of mediations presented by Zhao et al. (2010). For the statistical analyses, the package *lavaan* (Rosseel, 2012) for the statistical framework *R* (R Core Team, 2017) was adopted. Missing values with the scale items were below 1%. After testing for MCAR (χ^2^_(11)_ = 17.73; *p* = 0.09) according to Little (1988), we applied information maximum likelihood (FIML) to estimate missing values in the SEM.

### Method of the qualitative phase (Study 2)

As already addressed in Sect. 3.1, the qualitative strand was used to better understand significant and unexpected statistical findings. As the quantitative data revealed, distance learning during lockdown was associated with higher levels of vitality, although student teachers reported a significant decrease in fulfilment of the need for competence and relatedness as well as an increase in frustration of the need for competence. This finding was in contrast to expectations, and was further explored in the qualitative study.

The study followed a phenomenological approach to qualitative research. As Johnson and Christensen (2004) argue, “the key element of a phenomenological research study is that the researcher attempts to understand how people experience a phenomenon from the person’s own perspective” (p. 46). In order to get a more fine-grained and context-rich understanding of student teachers’ perceptions and experiences during the lockdown, in-depth interviews were applied.

#### Participants and interviews

A total of 14 student teachers (79% female; average age *M* = 22.86 years, *SD* = 3.76) enrolled in teacher training participated in the interview study. They did not take part in the quantitative survey, as the survey was carried out completely anonymised not allowing to contact students with a particular response pattern after the study. This may be regarded as a disadvantage in terms of design issues, as the quantitative and qualitative answers of the students could not be interwoven. However, selecting an independent sample of students for the qualitative part of the study may also come with several advantages. First and foremost, the student teachers of the qualitative part of the study were not influenced by answering the questionnaire items beforehand. In addition, the design assured complete anonymity of the student teachers during the whole study. Due to these reasons, we decided to choose an independent sample in the qualitative part of the study, but of course, the interviewed student teachers were from the same population in order to ensure comparability due to shared contextual conditions.

All interviews were carried out 12 weeks (first week of June 2020) after the sudden change towards distance learning via a video-conferencing tool due to safety measures. The interviews were conducted during the ongoing lockdown and distance-learning mode ensuring that the reflection of the student teachers on their experiences mirrored their current experiences in the lockdown. In doing so, retrospectivity, as a major limitation of (oral and written) self-reports, was reduced. Interviewees gave their consent for the recording of the conversations after being provided with written information regarding the researching interests and analysis procedure.

Besides follow-up questions for clarification and probing questions for elaboration, the interviewing procedure followed the interview guide to establish a high level of comparability of the cases during the analysis. The interviews were conducted by one member of the research team who had experience in interviewing and was an expert in the field in order to ensure investigator’s authority as a core quality criterion of qualitative research. Moreover, in order to enhance the trustworthiness of the collected data by reducing answers that are affected by social desirability, the student teachers were encouraged to speak frankly about their experiences prior to the interviews (for example, by assuring confidentiality and anonymity and pointing out that there are no right or wrong answers).

#### Data analysis

The audio material was transcribed verbatim with the help of a trained research assistant in accordance with the TiQ notation system that provides a standardised procedure on how to transcribe verbal data and is frequently used in qualitative research studies in the German speaking countries (Bohnsack, 2003). After reading the interviews in detail and writing short case summaries, a coding scheme was developed following structuring qualitative content analysis (Gläser-Zikuda et al., 2020; Mayring, 2014), resulting in a deductive-inductive coding scheme. The deductive categories were derived predominantly from the main research questions, whereas the inductive categories were developed directly from the interview material and specified the deductive main categories. Data analysis was supported by the software MAXQDA (VERBI Software, 2019). A section of the coding scheme is attached as "Appendix 2".

The coding scheme—particularly emerging inductive codes—was discussed among the researchers before the final coding scheme was determined and applied to the whole material. To check for intercoder-reliability as an indicator of the accuracy of the coding procedure, a second researcher coded five out of fourteen interviews. According to the procedure proposed by Brennan and Prediger (1981), a very good interrater-agreement for the developed coding scheme (κ = 0.83) and good reliability for the person-based analysis (κ = 0.73) could be achieved.

## Results of the quantitative phase (Study 1)

In the surveys, student teachers reported high levels of satisfaction for the needs of competence and relatedness, whereas satisfaction for autonomy was low. This pattern was the same before and after the lockdown. Regarding their frustration of BPNs, student teachers reported a high level of autonomy frustration, whereas the other means were significantly lower. In Table 2, both means for the first (before the pandemic policies) and second (during distance learning) samples are reported with a column for the mean difference. While satisfaction for competence and relatedness decreased, all other variables increased in the second sample with a remarkable increment (*Cohen’s d* =  + 0.34) for the vitality scale. Also, for the BPNSFS, we observed a meaningful difference regarding higher values of frustration for competence (*d* = + 0.28), whereas satisfaction for competence and relatedness were lower during the phase of distance learning (for both, *d* = − 0.30).

**Table 2:** Descriptives and correlation matrix

		*M*_t1_	*SD*_t1_	*M*_t2_	*SD*_t2_	*d*_Δt1-t2_	1	2	3	4	5	6	7
1. Satisfaction	Autonomy	3.24	0.73	3.21	0.87	− 0.05	–	*.66*	*.46*	− *.84*	− *.54*	− *.49*	*.47*
2	Competence	4.03	0.77	3.79	0.82	− 0.30	.55	–	*.45*	− *.49*	− *.90*	− *.51*	*.51*
3	Relatedness	3.90	0.85	3.64	0.88	− 0.30	.39	.35	–	− *.34*	*-.40*	− *.76*	*.28*
4. Frustration	Autonomy	3.52	0.93	3.60	1.00	+ 0.09	− .68	− .42	− .29	–	*.44*	*.45*	*-.48*
5	Competence	1.68	0.66	1.89	0.84	+ 0.28	− .44	− .76	− .32	.37	–	*.66*	− *.36*
6	Relatedness	1.66	0.66	1.73	0.73	+ 0.09	− .39	− .40	− .61	.37	.48	–	− *.30*
7. Vitality		4.09	1.29	4.55	1.47	+ 0.34	.41	.48	.24	− .43	− .34	− .25	–

*Coefficient above the diagonal and in italics refer to the correlation of latent variables; below the line, coefficients report the bivariate correlation matrix of the manifest variables.* *All correlations are significant at* *p* < .001

All variables of the *BPNSFS* reported significant correlations (*p* < 0.001) to the measure of vitality. The correlation matrix (see Table 2) indicates moderate to high coefficients for the satisfaction of autonomy (*r* = 0.41) and competence (*r* = 0.48) and the frustration of autonomy (*r* = − 0.43), whereas the other variables show smaller values (*r* = 0.24–0.34).

**Fig. 3:**
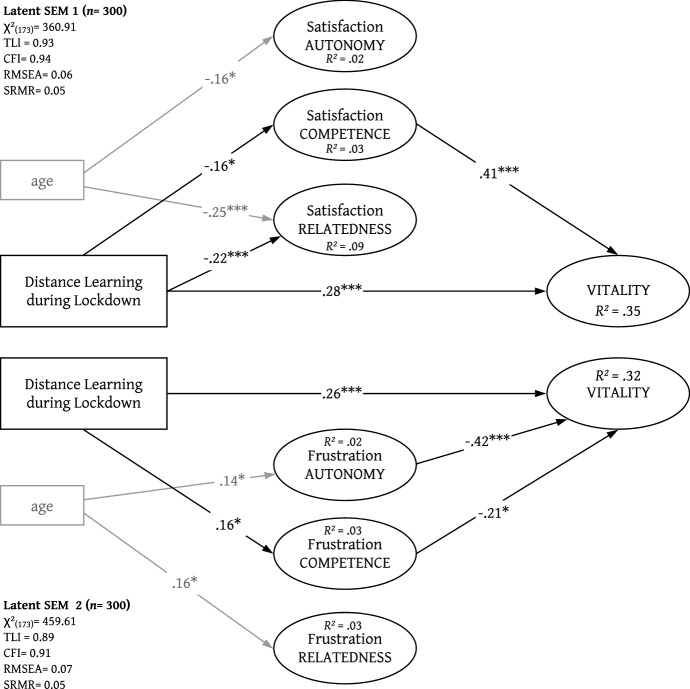
Structural equation model (SEM) indicating the standardised regression weights of the dichotomous variable (distance learning vs. face-to-face), need satisfaction and frustration on subjective vitality controlled for possible confounding effects by the age variable. All regressions and covariances were modelled, but only significant path weights are depicted for visual clarity. *p < 0.05, **p < 0.01, ***p < 0.001

To determine the relation between the variables of *BPNSFS* and vitality and assess their impact and interaction in the context of face-to-face vs. distance learning, we computed two latent SEMs (see Fig. 3).

In contrast to the conceptual model (see Fig. 1), we had to compute two separate SEMs due to high collinearity between some latent variables of the *BPNSFS* (e.g. between satisfaction and frustration of the need of competence [*r* = 0.90, *p* < 0.001]), which resulted in misleading standardised beta weights larger than one (Piedmont, 2014). Since an aggregated solution of the *BPNSFS* was not possible due to poor model fit, we opted to estimate two separate models, one for the satisfaction and one for the frustration of *BPN*. Both models reflected a good fit (Brown, 2006): SEM 1 χ^2^(173) = 360.91, TLI = 0.93, CFI = 0.94, RMSEA = 0.06, SRMR = 0.05; SEM 2 χ^2^(173) = 459.61, TLI = 0.89, CFI = 0.91, RMSEA = 0.07, SRMR = 0.05.

In SEM 1 (see Fig. 3), both satisfaction of BPN and the measurement during distance learning proved to be a significant predictor of vitality. Satisfaction of the need for competence (*β* = 0.41, *p* < 0.001) was linked to a higher perception of vitality. A similar result was found for the dichotomous variable of distance learning, which reported a positive relation with vitality (β = 0.28, *p* < 0.001). On the contrary, distance learning during lockdown was also associated with a lower satisfaction for the need for relatedness (β = − 0.22, *p* < 0.001) and competence (β = -0.16, *p* < 0.05). The second model (SEM 2), which included frustration of BPN, presented an analogous picture: Both frustration of autonomy (*β* = − 0.42, *p* < 0.001) and competence (β = − 0.21, *p* < 0.05) were linked to a lower perception of vitality. Distance learning during lockdown was again a predictor for higher vitality (β = 0.26, *p* < 0.001), but also contributed to higher frustration for the need of competence (β = 0.16, *p* < 0.05). In both models, we implemented age as a covariate to control for possible sampling effects: Although age was not linked to the perception of vitality, it showed significant paths to BPNSFS.

By testing all relationships for mediation effects, we observed one competitive mediation effect (Zhao et al., 2010) despite low impact and robustness. The effect of distance learning on vitality was partially mediated by the satisfaction of need for competence (β_indirect_ = − 0.06, *p* < 0.05), which resulted in a slightly lower total effect (β_total_ = 0.22, *p* < 0.001) compared to the direct (β_direct_ = 0.28, *p* < 0.001) path of the predictor.

Overall, the SEMs explained between 32 and 35% of the variance for the outcome variable.

### Brief discussion of the quantitative phase (Study 1)

Contrary to our conceptual model (see Sect. 2.1.1), distance learning during lockdown was associated with higher levels of vitality. In contrast, this period was linked to lower satisfaction of relatedness and competence and an increase in the frustration of the need for competence. Both the satisfaction and frustration of competence and the frustration of autonomy were significantly related to student teachers’ perceived vitality, which is in line with previous findings (Martela & Sheldon, 2019; Ryan et al., 2010). A possible explanation for the unexpected result could originate in the need for autonomy, which was not significantly related to whether student teachers studied under normal or lockdown conditions. Furthermore, only the frustration of the need for autonomy was linked to perceived vitality. It could be assumed that some student teachers may have experienced a greater flexibility in their daily structure in the first lockdown, which has been reported to be linked to higher vitality (Ryan et al., 2010; Smolders et al., 2013), whereas others may have experienced this period as rather dissatisfying or need thwarting regarding perceived autonomy. To explore this assumption, the results of the qualitative study were of utmost importance as they allowed an in-depth understanding of the student teachers’ experiences during the lockdown.

## Results of the qualitative phase (Study 2)

This section starts with an analysis of person-based changes during the lockdown in order to illuminate experienced changes in vitality and psychological need-fulfillment on a person-level. After that, the results are presented around the three main themes that were derived deductively from the research questions: (1) vitality and its influencing factors before the pandemic; (2) changes experienced during the pandemic and its association with vitality; (3) fulfillment and thwart of the need for competence during the pandemic.

### Person-based changes

We undertook a person-based perspective and analysed the qualitative data by coding each interview regarding changes of vitality and competence (see Table 3). This procedure made it possible to compare whether an increase in vitality and a decrease in the fulfilment of the need for competence was also observed in the qualitative sample in order to cross-validate the findings based on different methodological approaches. The results point out, that the majority of student teachers reported a strong (+ +) or moderate ( +) increment for perceived vitality during distance learning and lockdown measures. However, some reported an initial drop in vitality and an increase after some weeks (−|+) and a few reported a decrease (−). Half of the interviewed participants indicated an attenuated (−) or significantly reduced (− −) perceived competence for the corona term, whereas others reported either a moderate increment (+) or no difference in comparison to a regular term. Thus, the findings of the qualitative person-centred analysis are mostly consistent with the results detected in the quantitative strand of the study. The question that follows: What factors influence this change in vitality and perceived competence? This question will be addressed in the following sections.

**Table 3:** A profile-based analysis of the qualitative interviews concerning changes in the perceived vitality and competence during distance learning and lockdown measures

#interview	age and gender	Δ vitality	Δ competence
1	31 m	+ +	=
2	22 f	+ +	+
3	20 f	+	− −
4	28 f	+ +	=
5	21 f	+ +	− −
6	21 f	+ +	− −
7	21 f	+	+
8	21 f	+ +	+
9	30 m	− |+	− −
10	21 f	− |+	− −
11	21 f	−	−
12	20 f	−	+
13	20 f	− |+	=
14	21 m	+	−
	79% female	64% + or + +	50%—or—-
	*M* = 22.86 years	21%—|+	29% +
	(*SD* = 3.76)	14% -	21% =

### Vitality in the context of university and private spheres

In order to be able to accurately frame changes of vitality during the pandemic, we first need to get an in -depth understanding of student teachers perceived vitality and its antecedents during regular conditions. Thus, all interviews commenced with a characterisation of vitality for private and study-based contexts with reference to a regular semester. Questions concerning both fostering and hindering elements for the experience of vitality were asked for explicitness. For a regular semester of study, both private and university-based contexts were identified as sources for increased experiences of vitality. The resource most mentioned was relatedness towards fellow student teachers, which was mentioned both for in-class activities, as the cooperative and interactive learning formats, as well as for private situations between the university courses.

But also just <um> <um> working together in a group. Also concerning a thirst for knowledge, being able to exchange ideas. Yes, exchanging ideas. (I13; #00:02:11-9#) <Hmm>. Normally, always when doing something with others. Like when walking from one building to another together; talking to each other and in doing so one chats and laughs and has fun. When going for lunch in the canteen together at the university or elsewhere, just really always when meeting OUTSIDE the lectures. (I10, #00:00:33-0#)

The experience of vitality for students can differ depending on how instructors design the learning experience within each course. For example, interactive and physically stimulating seminars, topics that are proposed in an interesting way and content that is perceived to be meaningful are more likely to be associated with vitality. Also, the feeling of relatedness to lectures (having opportunities to discuss, interact with them and perceiving the lectures to be acknowledging about personal conditions) leads to higher vitality.

For the private context, the main sources of vitality are linked to leisure time and physical activity. Some student teachers explicitly refer to these activities as being compensatory for a rather lethargic day of attending university courses:

[…] <Um> it depends very much on the balance. For example, when having felt lethargic and bored at university, then as a result, it happens to me that when I am at home, […] this energy that I have not yet used (.) suddenly has to be acknowledged and used up. Whether it be through playing volleyball, running or fitness. […] (I14; #00:04:44-0#)

Social interactions outside the university were also mentioned as sources for vitality as well as the satisfaction of physiological needs, such as sleep, food and hydration.

On the other hand, elements hindering the perception of vitality were related mainly to the context of the university. Besides singular mentions of physical impairment, personal problems and few opportunities for physical activities, interviewees predominantly pointed to the study programme as being responsible for their reduced vitality.

Student teachers identified the time anticipating and completing the exam period as the main challenge for the experience of vitality. Although some mentioned pressure due to assignments during the semester, many described how their vitality peaked at the beginning of the semester, after which they experienced a gradual decrease to its lowest level during the exam period.

Hm (.) I think (.) in general I have to say that I experienced more vitality at the beginning of term because at that time I was just more motivated, and then slowly after the summer holidays I was naturally like very relaxed, I would say, and then closer to the end and especially during the exam weeks it became increasingly less so (.) I just noticed that (.) I started the day motivated and (.) did not feel tired and worn out; towards the end that rather happened. Then I hardly got out of bed, I have to say, and I noticed that my concentration really slowed down and that I could not remember as much as normal when learning. (I06, #00:00:51-7#)

During the semester, vitality can be lower if the workload (e.g. number of elective courses) was set too high or the burden of both occasional jobs and studies caused tiredness. Poor scheduling of course tables (resulting in dead time spent inefficiently), low relevance of course content and passive participation in frontal instruction resulted in reduced vitality:

Like when, for example, in a lecture, when a professor is currently just standing at the front and talks and talks all the time, then I really hit rock bottom with my energy. (I13, #00:02:43-5#")

**Fig. 4:**
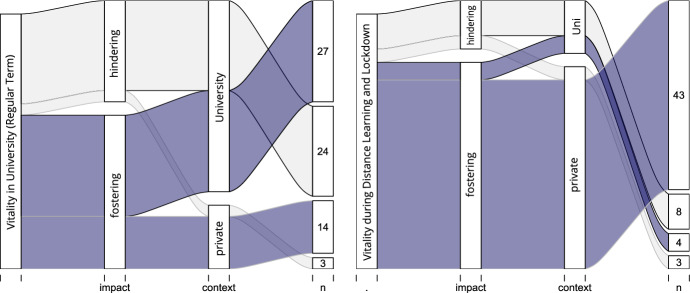
Alluvial diagrams reporting the frequencies of the deductive categories of the coding system for Themes 1 and 2 (see Sect. 5) of the qualitative interviews

In Fig. 4, we summarise and illustrate the interview codes, which indicated a different characterisation of vitality between a regular study term and distance learning and lockdown. By using an alluvial diagram, we were able to identify a distinct shift regarding the impacts of the contexts (study vs. private) before and after the lockdown.

In a regular term, references to subjective vitality were mainly linked to elements situated in the context of the university, and based on the frequencies, they were mostly balanced between fostering and hindering effects. In contrast, after the lockdown, the focus turned towards the private context. As many student teachers reported, they found themselves back in their hometowns and villages with their families. Additionally, online-based learning increased their autonomy in scheduling their time and it reduced the common dead time between classes and when commuting to and between campus buildings. Likewise, the private context was connected with elements fostering vitality, such as relatedness and nature, whereas the references to hindering causes were mainly discussed in relation to the university context.

### Changes due to lockdown measures and distance learning

In the second part, the interviews focused on the changes due to the lockdown measures and their experience of vitality during the phase of distance learning. In half of the interviews, student teachers mentioned how the lockdown measures of the government resulted in the sudden shift towards distance learning (communicated by email and implemented within one afternoon) and a radical change in their basic personal routines. Several participants reported how they moved back to their families on the same day, which meant those used to living in student dorms or shared apartments with other students were now spending significantly more time with their family. On the other hand, those who did not move back to their hometowns reported feeling separated from friends and family members for several weeks. Additionally, some missed the possibility to carry on routine activities, such as their student job or church attendance, while experiencing an overall deceleration of the day.

The main changes reported were linked to the context of their studies. Although changes in this regard were perceived as rather negative, some positive dimensions were reported as well. At first, several student teachers emphasised the uncertainty and the lack of information coming from some lecturers who adapted too slowly or not at all to the new teaching format:

[…] <um> but for me in the beginning it was a big adjustment that some professors just did not communicate, and no one knew how to proceed and what is going on. (I13, #00:04:16-5#)

The most mentioned negative aspect regarding distance learning was the (partially) poor support provided by lecturers in their online-based teaching. Such comments fell into one of two dimensions: On the one hand, several courses did not provide for a webinar-based or other interactive format but rather implemented a format based on written-task assignment and reading of textbooks or scripts:

Well, it is like, you have to learn everything yourself, now even more, just because mostly hmm the explanations from the professors are missing and often they just say: “Read this and solve it!”. And that makes it much harder, when you don’t have a clue, you don’t get any input and all you are doing is reading. Because if a teacher does explain something, then you process it differently than when reading it yourself. (I12, #00:03:43-8#)

On the other hand, students missed the personal contact and the possibility to interact with lecturers. This reduced both their feelings of belonging and being part of the course. Further, the new distance learning format affected their sense of competence because they lacked opportunities to ask clarifying and follow-up questions or receive feedback from the lecturer:

So I have a few courses with no not a single online meeting till today and they just give you a long list what you have to do, but you have (.) no idea at all what the professor sounds like or (.) what he wants from you, so to say (.) and that was just - so a few did a great job, I think. With them I really, like I felt I really am part of the course. And with a few others that was not even remotely the case. (I06, #00:03:47-7#)

[…] another big problem is, asking questions, if one did not understand, that is a big problem, because you always have to do that via email. And you cannot just think, OK – I’ll wait for the next lecture (.) and then I’ll just ask. (I14, #00:10:03-3#)

Some student teachers mentioned how the lack of interaction and guidance from the lecturers was partially compensated by the social support between students. For example, online-based study groups and the sharing of relevant information helped them to stay on track and current with the lecture material. However, the interaction between students in terms of formal, content-related and informal discussions, was an aspect student teachers missed. They felt such interaction was not of the same quality when using web-conferencing platforms and referred to the overwhelming amount of screen time, technical difficulties and confusion linked to a large number of different e-learning tools as additional negative characteristics of distance learning.

So, if the change to distance learning was linked with so many negative outcomes, how can we explain the increase in vitality?

First, student teachers reported some lecturers adapted quickly and incorporated interactive web-based formats, which resulted in sufficient interaction with students and teachers and to additional information and communication technology (ICT) competencies. Furthermore, effective web-based teaching allowed for more self-regulated learning and, in consequence, resulted in more efficient planning of study time. Almost half of the students referred to higher levels of autonomy regarding their planning of study activities better suited to their preferred diurnal cycles:

<um> in the end you @get up 10 minutes before@, get your coffee and then sit down and then solve the tasks that are in the course and so. That is the difference, I felt more awake than like in the courses that started at 9am. <Um> (2) yes and in general … that in the end I had the chance to schedule and manage my tasks and assignments better because the deadline was in a couple of weeks, there were later deadlines and not those rather tight turnaround times (.) like as it was before. (I02, #00:06:42-5#)

The higher degree of autonomy as a result of asynchronous teaching and learning led some students to a more efficient approach to studying and a reduced feeling of stress and pressure due to high workload and short deadlines. For many, this meant they had more time at their disposal for individual activities.

<um> yes I would say that. So, (.) you can nonetheless manage your things better now, you had (.) <um> in general the change to decide: “When do I do what?”. And then it is partly less stressful, because one could see, that now there is time to do it and in a normal term, I would have to attend all these courses and now some were cancelled (.) because there was nothing online. And then you have way more hours, more time and you can manage it better. That (2) is something I rather approve of. (I03, #00:06:19-7#)

Nevertheless, the increase in vitality noted during distance learning was mostly not linked to the context of the university and learning directly but mentioned in regard to the increase of spare time and one’s private live:

Increased autonomy and time available for other activities led students to spend more time participating in physical activities, which were mainly outdoor and in nature. Several students reported they intensified or started to exercise and play sports regularly during the lockdown, as distance learning provided more flexibility:

And I just did more sports than usually. So that is something I noticed that was good for me. Then I also experience more vitality and I can better concentrate on my studies when I do one hour of sport in the morning and only AFTER THAT do I sit down at my desk. (I10, #00:04:41-5#)

Several students reported how hiking in nature or spending time in the garden was a great source for feeling vital and energised during the lockdown. Also, some students reported more outdoor time combined with more time spent with family, which had been reported as an additional element in fostering vitality.

And I had a bit of time to go outside. The weather was often very good I have to say. We spent a lot of time outside – also with my family. And that really helped me a lot. Just being able to lie in the garden all day long, then you are more motivated the next day and you experience more vitality. So, for me it was easier to experience more vitality than during (.) normal university. So with all the face-to-face lectures. (I06, #00:04:39-5#)

[…] If you do not live on your own, for example, then you can go outside with people (.) you share a household with. So I went hiking a lot, went for walks and these things, spent much more time in nature. […] (I07, #00:04:20-1#)

Since social distancing permitted close contact with only members of the same household for the first two weeks, students’ connections with family members and flatmates or partners were intensified, whereas digital tools were utilised to maintain contact with friends and colleagues. Both dimensions of social relatedness were referred to as conducive for vitality.

On the individual level, vitality was increased by new opportunities for pursuing and (re)discovering (new) hobbies. Furthermore, better sleep quality and more mindfulness contributed to positive outcomes during the lockdown. Concerning this, some mentioned how distance learning made it necessary to increase personal boundaries regarding their work-life balance, as personal space also became study space.

Concluding, although many students reported some negative changes in connection with their studies (e.g., little support by the instructors; technical problems), the increase in autonomy and time available for non-academic activities (e.g. spending time in the nature or with family) led to an increase in vitality suggesting a compensatory mechanism by putting more emphasis on private matters compared to the academic context in order to increase one’s perceived vitality. More concretely, a key result is the increase in physical activities and in time spent outdoor based on student’s higher degree of flexibility in regard to the study time table. Likewise, a better fit to the diurnal cycles and the possibility to engage in recreational activities contributed to more perceived vitality. Nevertheless, the results also allow the conclusion that a high-quality online learning environment (e.g. regular contact with the instructor and the fellow students) did also contribute to a balanced experience of vitality.

### Changes in competence

Finally, following the result of the quantitative study, in which a significant decrease in the need for competence was observed, we asked the student teachers about changes they experienced in terms of competence during the lockdown semester.

On an individual level (see Table 3), many students experienced a decrease in satisfaction of perceived competence, whereas others reported an increase or no perceived differences compared with other semesters. Student teachers who reported an increase in competence described how web-conferencing allowed for interaction with the teacher and other students and a more individualised learning experience. For example, some noted they appreciated being offered meaningful choices for topics assigned to the in-class presentations:

[…] not having to work on a fixed topic but reach out for your own topic to share with others. So, somehow, there was more attention given to individuality. And, I find that’s very important. And <um> one just feels more competent concerning that. (.) <um> if you can select the topic yourself and you can explain it and, in the end, help them to understand it. And, I mean, I am studying to be a secondary school teacher (.) so (.) of course that suits me more and I also feel more competent. And (.) because it is exactly in this direction that I am heading. (2) yes (I02, #00:09:36-5#)

For some, the experience of self-instruction and self-regulated learning resulted in higher levels of self-efficacy. Despite initial struggles or setbacks, some student teachers reported feeling more confident about their skills and their competence acquired by higher personal dedication to the course content, as one student explained:

Right from the beginning I was overstretched with all the assignments and trying to establish a system, somehow but meanwhile it works really well and that has shown me personally that I can also do it on my own – even if I have to learn it myself. And (.) it did show me that I find what I do interesting and it confirmed to me that I have chosen the right programme of study – and future job. And it confirmed – that it is good for me. (I08; #00:06:22-0#)

For others, the higher degree of autonomy, partially linked to more flexible deadlines for work assignments, was an additional source for increased competence because it allowed for a more focused and deeper learning process.

Conversely, those who reported a decrease in competence mainly referred to the high degree of self-instruction – often in absence of further support from the lecturers or due to a poor didactical concept of the course teaching. According to some responses, this was especially the case for courses in which lecturers referred to a textbook or a script as the main body of content for the exam. Similarly, this was reported for those professors who used technology to stream their lectures in which they mainly read their materials out loud or recorded their lectures without any meaningful didactical adaptions, which would have been necessary due to the mandatory distance learning and the unusual learning circumstances.

[…] So (2) there were {subject} lectures that continued more or less like every other term – only with less detailed explanations, where the script was only read through - read out loud and there was even less time, normally you take at least notes and there is writing on the board, but this way you were only read out what was already in the material. […] (I11, #00:08:22-9#)

Also, some assignment-based formats of distance learning led to a lower feeling of competence as a result of a lack of engagement and low support from teachers. Some students recalled completing several assignments without a perception of development or progress regarding the course outcomes.

So, before I had the feeling that I learnt something during term, to get to know something new. Now that is often not the case. Now I always say, “Ok, I do the task, but I don’t take anything from it” (I10, #00:06:52-6#).

Yeah. So now like in {subject} that is one of my subjects, there is one course, there we just got the worksheets and I do not have the feeling right now that filling in words and so on contributed to my development. So what I was missing was (.) a direct feedback somehow. (I13, #00:07:19-1#)

In sum and from the perspective of student teachers, reduced levels of competence were linked mainly to the initial uncertainty of the course organisation and to inadequate didactical concepts for the mandatory distance learning. These results suggest—in line with the results on students’ vitality during the pandemic—that a high-quality (distance-based) instruction during the pandemic does indeed significantly matter for student teachers’ psychological functioning with regard to their studies.

### Brief discussion of the qualitative phase (Study 2)

The qualitative phase aimed to understand the changes in vitality by investing the antecedents with a special emphasis on the need for competence, which—in both quantitative models—was linked with the subjective vitality and decreased after the lockdown. The person-based analysis revealed that a qualified majority described a (rather) strong increase in vitality during the first lockdown. In the qualitative thematic analysis, this change was explained by greater flexibility in the day structure, increased physical activity, and more time spent outdoors in the nature which has already been observed as a source of vitality in previous research (Ryan et al., 2008, 2010; Smolders et al., 2013). In contrast, about half of the student teachers reported a decrease in perceived competence which they traced back to a poor adaptation to the distance mode, little or no feedback from lecturers on the learning process, and uncertainty whether they will succeed in the courses. Nonetheless, the other half of student teachers reported no changes or an increase in perceived competence as, for example, the new study mode allowed for a more autonomous and self-directed study mode. Both results underline that online-based study modes can be improved by considering the BPN of students (Murphy, 2020; Wang et al., 2019).

## General discussion

In this study, we applied an explanatory sequential mixed-methods design to understand the difference regarding the experience of vitality and one’s satisfaction and frustration of BPNs between a regular university term and during a period of distance learning due to severe COVID-19 lockdown measures in Austria. To this end, we first computed an SEM to determine both the impact of the study context and the mediation effects of BPNs on the experience of vitality. Our hypotheses were only supported in part: Studying by distance learning during lockdown measures was associated with lower satisfaction regarding the needs of relatedness and competence (H_1_). Likewise, the corona term was linked to greater levels of frustration of the need for competence (H_1_), whereas frustration concerning both the needs for autonomy and competence was associated with lower vitality. For this, the positive impact of distance learning during the lockdown resulted in a competitive mediation effect (Zhao et al., 2010) through reduced levels of the need for competence (H_3_).

According to the conceptual model, we expected a significant decrease in subjective vitality (H_2_) as a result of lower satisfaction and increased frustration of BPNs due to the major changes linked to distance learning and the challenges for self-regulation during lockdown measures. In particular, reduced contact with peers and lecturers, decreased active participation due to digital learning formats and reduced mobility and opportunities for leisure time activities were expected to have a detrimental impact on the students’ vitality and BPNs (Aldrup et al., 2017; Ryan & Deci, 2008; Wang et al., 2019). Consequently, we were only able to confirm H_1_ and H_3_ (see Sect. 2.1.1).

In line with previous research, vitality was associated with the satisfaction of BPNs (Martela & Sheldon, 2019; Vansteenkiste et al., 2020). The competitive meditation through the need for competence exemplifies how BPNs “can also play a buffering role against the emergence of malfunctioning through helping to build inner resources that contribute to subsequent coping” (Vansteenkiste & Ryan, 2013, p. 265).

In contrast, the significant increase of vitality observed during the lockdown was not in line with our expectations. Furthermore, we detected significant lower levels of competence during the mode of distance learning. These results led us to investigate further the rationale behind the increase in vitality during distance learning in lockdown using qualitative in-depth interviews and clarify if and how perceived competence changed during the corona term.

In the interviews, students often reported how their relatedness to fellow students decreased during the lockdown, which would likely have resulted in lower vitality. Alternatively, the deceleration of activities, a better fit to the diurnal cycles and the increase of spare time might have contributed to higher vitality values, which could explain the unexpected quantitative findings. Relevant was also the increase in physical activity and the possibility to spend time in nature.^1^ The increase in possibilities to experience relatedness to family members and romantic partners, participate in physical activities and spend time outdoors (Ryan et al., 2010) can explain why student teachers experienced more subjective vitality although reporting decreased psychological needs satisfaction for the learning environment. In the interviews, student teachers reported a greater desire to spend time in nature,^2^ which is associated with leisure and recreation. Being in nature also increases exposure to daylight, which may also be a physical factor contributing to enhancing vitality (Smolders et al., 2013). The energising effects of exposure to nature on vitality correspond with diary-based research (Ryan et al., 2010) and are likely responsible for the increase in vitality during lockdown despite reduced needs satisfaction.

Conversely, both of our studies emphasised the reduced satisfaction of perceived academic competence, which is a negative effect of the changed study mode (for some courses only rudimentarily improvised) during lockdown. Data were collected in the middle of the most severe lockdown in Austria. To complicate matters, student teachers did not know how long they would have to study in distance mode and benchmarks for (distance) exams were not available at the time. Lecturers with varying levels of experience concerning web-based learning had to alter their courses with little notice from one day to another (Kanuka et al., 2008). Many student teachers also underwent practical training and had to negotiate alternative ways to complete their practical phases. In other words, the structure they were used to when studying at university was shattered, and this might have resulted in less satisfaction and more frustration of the need for competence (Reeve, 2018). Student teachers also reported in both studies that online formats and the restricted possibilities to socialise with fellow students from the university could not compete with the feeling of relatedness in a normal term, stressing the importance of informal contacts at university for social relatedness (Ryan & Deci, 2017).

### Limitations and future directions

As with any study, the present study comes with certain limitations. One methodological limitation to both phases of the study is that the absence of an experimental design means we could not infer causality. Additionally, the implemented sampling method consisting of two measurements of the same cohort of student teachers (student teachers at an Austrian university at the end of their bachelor’s or beginning of their master’s degree) is less robust in comparison to a true longitudinal panel design. These student teachers participated in the study on a voluntary basis. Since a random-sampling strategy could not be applied, covariates were controlled to avoid possible sampling bias. The strength of the SEM analysis and the robustness of the findings were confirmed by the qualitative research, which validated the impact of the independent and mediating variables on the outcome variable and illustrated the interaction between the (changes of the) learning context and subjective vitality.

An additional methodological limitation concerns the rejection of the conceptual model when computing the SEMs, as issues concerning multicollinearity of the mediating variables did not allow for a conjunct estimation of both the frustration and the satisfaction of the needs (Shieh, 2006). After careful deliberation, we opted for two separated models, as the factor analysis confirmed the postulated and previously validated six-dimensional structure (Heissel et al., 2018) and the preliminary models with all six mediating variables reported higher levels of the coefficient of determination (*R*^2^). This means the variables should not be rejected due to a possible redundancy as they—despite substantial levels of covariance—provide an added empirical and theoretical value to the study results (Paulhus et al., 2004; Shieh, 2006).

Furthermore, we must also consider that this study did not include socio-economic variables. During the interviews, student teachers reported supportive (family) environments. However, other publications have reported several negative impacts of the lockdown on the well-being of students, for example, financial insecurities due to a job loss and mental-health problems (Fawaz et al., 2021; Kumar & Nayar, 2020). Accordingly, the findings of our study must be considered with caution to avoid misleading conclusions and practical implications.

## Conclusion and implications

The results of this study help to better understand the vitality of student teachers and identify detrimental as well as supporting factors. Understanding how to facilitate vitality will help both students and faculty face new challenges in regard to the ongoing pandemic as well as ongoing developments towards an increase in online-based teaching in university courses (Uerz et al., 2018). In particular, this research stresses the possibilities to positively influence BPNs to anticipate optimal outcomes related to academic and personal development. As this research shows, it is crucial to foster opportunities to interact with the learning environment and increase relatedness with fellow students and lecturers when designing distance learning formations. Although this conclusion is not new to the literature on online-based education (e.g. Butz & Stupnitzky, 2017), it is likely to receive a particular accentuation during a pandemic when social contacts are not only restricted in the academic context but as a whole. An emphasis on interactive elements in instruction fostering the sense of relatedness and competence alike may be particularly supportive in such challenging times. Likewise, our results emphasise the importance of giving and receiving formative feedback to support the learning process (Uribe & Vaughan, 2017), especially when the immediacy of the in-class situation is limited by a web-conferencing or task-based asynchronous teaching design. Nevertheless, even if most teacher educators and student teachers hope for a quick return to face-to-face settings, it is likely that the shift towards distance learning has produced a significant boost in ICT competencies for teacher educators and student teachers. This increase in skill will impact further development of digital skills necessary to face the challenges of the current century as already addressed in several competence frameworks (e.g. The European Framework for the Digital Competence of Educators; Caena & Redecker, 2019). It remains to be seen how teacher educators will use these digital skills in their future teaching once the pandemic is over. As teacher educators are important role models for future teachers (Lunenberg et al., 2007), it is hoped that these acquired skills will be further used and, above all, built upon in a continuous process of professional development.

## Data Availability

The datasets generated during and analysed in the current study are not publicly available due to further, ongoing research projects but are available from the corresponding author on reasonable request.

## Appendix 1: Interview guide

**Table:**

#	Question
0.	Greeting and information
1.	In this study, we would like to investigate the topic of vitality. The experience of vitality is described as a holistic functioning associated with physical and psychological well-being; the feeling of being activated and able to mobilise yourself
	On this slide, I can show you some items that were used to assess “vitality” [a slide is shown via the web conferencing tool]
2.	Now, think about a “regular” semester at university, perhaps the winter semester or last years’ summer semester:
	How and when did you experience vitality at university and in your private life? How does it manifest itself?
	*Possible follow-up question:*
	On the other hand: What hindered you from feeling energised or able to mobilise yourself? What might have drained your vitality, so to speak?
3.	Let’s move on to the current semester: In mid-March, the shift due to the Corona measures were implemented quite unexpectedly
	What were the major changes for you in your private life as well as in your studies?
	*Possible follow-up question:*
	How did studying in the teacher training programme change during the "Corona Semester"?
4.	Were you still able to feel vital and active during the "Corona semester", despite, or even because of, the restrictions?
	Possible follow-up question:
	Did you discover new ways to feel vital or energised by the new circumstances?
5.	[As you’ve already mentioned], we experience ourselves as active and energised during our studies when we also feel competent; that is, when we can fulfil study-related expectations
	In your perception, did your experience of competence change over the “Corona semester”?

## Appendix 2 Section of the code scheme based on a structuring qualitative content analysis

**Table:**

5 Perceived Vitality (Q4)	5.1 Rationale for no change in perceived vitality	
	5.1.1 Workload / study approach unchanged	
	5.2 reducing factors	5.2.1 Reduced exam activity
		Description:* Anticipation that this semester fewer courses will be completed.*
		Reference example: Probably, uh (.) I won’t be able to successfully complete or pass my final exams and my course assignments. (.) <um> yeah. So, I feel a little less competent (.) that I will master it. (I 14, #00:14:38-2#)
		5.2.2 Lack of relatedness
		Description:* Reduced perceived competence due to missing interaction with lecturers and students as communication in class (e.g. participating in discussions) is limited.*
		Reference example: Yes, definitely. So, I am much more incompetent. Because - with the personal contact -, I think I already said that-, so I think (.) I can demonstrate my strengths much better, or so to speak, show the lecturers how motivated I actually am and how much I enjoy being there and that I like taking the class […] (I09, #00:10:37-0#)
		5.2.3 Lack of self-regulation
		Description:* Facing difficulties with regard to self-regulation (self-motivation, self-assurance, self-management/work organization etc.)*
		Reference example: Because then [studying in distance learning] you might also be a bit negligent, because (.) you're at home. And for me – it has always been like that - I've always done everything in {city of the University} and then made sure that I have a lot of time for leisure and for myself in {native city}. Now, that is no longer the case. Now, there is no longer a separation, at all and (.). That was a bit more difficult for me because at home, at home in {native city}, I had to motivate myself and that was rather difficult for me at the beginning, I must say. (I05, #00:07:53-0#)
		5.2.4 High proportion of self-study- lack of/reduced guidance
		Description:* Reduced perceived competence in courses with mainly self-study mode – with emphasize on poorly structured materials and no additional resources from lecturers.*
		Reference example: […] So (2) there were {subject} lectures that continued more or less like every other term – only with less detailed explanations, where the script was only read through - read out loud and there was even less time, normally you take at least notes and there is writing on the board, but this way you were only read out what was already in the material. […] (I11, #00:08:22-9#)
		5.2.5 Increased examination pressure at the end of the semester
		Description:* Exam-related pressure to complete all sections of the course syllabus.*
		Reference example: The only thing that is more difficult is the examination phase. Because due to all the Corona measures, for many lectures it was not very clear from the beginning (.) what the exam will look like. So now, many things have been pushed to the end of the semester (.) so now, everyone is under stress. I think the exam phase is a bit worse now, because the content of the exam will be like the one from a regular semester. (I07, #00:07:06-0#)
	5.3 incrementing factors	5.3.1 Supervised study mode – Synchronous lectures
		Description:* Web-based, synchronous lectures with higher interaction with lectures provide a feeling of “staying on track” and learning growth.*
		Reference example: But there are other subjects where you have already had online courses […] where you have the feeling that you can contribute to the whole thing yourself. You are not only dependent on a few work sheets (.) and yes, in these subjects you are more optimistic that you will pass the course. (I13, #00:07:19-1#)
		5.3.2 Increased autonomy / flexibility on task and assignments
		Description:* Higher degrees of autonomy linked to meaningful choices of presentation for assignment topics as well as flexibility on the study pace and deadlines. *
		Reference example: […] So, somehow, there was more attention given to individuality. And, I find that’s very important. And <um> one just feels more competent concerning that. (.) <um> if you can select the topic yourself and you can explain it and, in the end, help them to understand it. […](I02, #00:09:36-5#)
		5.3.3 Adjustment of criteria and requirements ("scaling down")
		Description:* Demands and course requirements were adjusted and allowed for broader exploration and individual approaches on assignments*
		Reference example: Occasionally, expectations have been lowered (.) <um> and so you could try out more what you always wanted to try without being afraid that you would fail the course (I02, #00:09:36-5#)
		5.3.4 Experience of self-efficacy linked with self-regulated learning
		Description:* The initial struggle with the distance-learning mode resulted in higher levels of self-efficacy following the experience of successfully completing task and assignments.*
		Reference example: Right from the beginning I was overstretched with all the assignments and trying to establish a system, somehow but meanwhile it works really well and that has shown me personally that I can also do it on my own—even if I have to learn it myself. And (.) it did show me that I find what I do interesting and it confirmed to me that I have chosen the right programme of study—and future job. And it confirmed—that it is good for me. (I08; #00:06:22-0#)

## Footnotes

1 During the most severe phase of the lockdown in March and April 2020 (timing of the second measurement), the Austrian population was only allowed to leave their homes for three main reasons: grocery shopping, work obligations and assistance to others. However, the government encouraged citizens to spend time outdoors if such recreational activities were participated in alone or with one single member of the same household (BGBl., 98/2020).

2 During some of the online-based classes, nature sounds, such as twittering birds, were heard through the audio signal of some students who participated in the web-conferencing while sitting with their devices in their gardens.

## References

[CR1] Aldrup K, Klusmann U, Lüdtke O (2017). Does basic need satisfaction mediate the link between stress exposure and well-being? A diary study among beginning teachers. Learning and Instruction.

[CR2] Bartholomew KJ, Ntoumanis N, Ryan RM, Thøgersen-Ntoumani C (2011). Psychological need thwarting in the sport context: Assessing the darker side of athletic experience. Journal of Sport & Exercise Psychology.

[CR3] Behzadnia B, FatahModares S (2020). Basic psychological need-satisfying activities during the COVID-19 outbreak. Applied Psychology: Health and Well-Being.

[CR4] Benyamini Y, Idler EL, Leventhal H, Leventhal EA (2000). Positive affect and function as influences on self-assessments of health: Expanding our view beyond illness and disability. The Journals of Gerontology.

[CR5] BGBl. (2020). Verordnung gemäß §2 Z 1 des COVID-19-Maßnahmengesetzes [Regulation according to the COVID-19-Measures Act] Federal Ministry of Social Affairs, Health, Care and Consumer Protection. Accessed July 06, 2020. https://www.ris.bka.gv.at/eli/bgbl/II/2020/98/20200315.

[CR6] Bohnsack, R. (2003). *Rekonstruktive Sozialforschung: [Reconstructive Social Research.]* (5th ed.). Budrich.

[CR7] Brennan RL, Prediger DJ (1981). Coefficient Kappa: Some uses, misuses, and alternatives. Educational and Psychological Measurement.

[CR8] Brown, T. A. (2006). *Confirmatory factor analysis for applied research*. Guilford Press.

[CR9] Bryman, A. (2007). Why do researchers integrate/combine/mesh/blend/mix/merge/fuse quantitative and qualitative research? In M. M. Bergman (Ed.), *Advances in mixed methods research* (pp. 87–100). Sage.

[CR10] Butz NT, Stupnisky RH (2017). Improving student relatedness through an online discussion intervention: The application of self-determination theory in synchronous hybrid programs. Computers & Education.

[CR11] Caena F, Redecker C (2019). Aligning teacher competence frameworks to 21^st^ century challenges: The case for the European Digital Competence Framework for Educators. European Journal of Education.

[CR12] Cantarero K, van Tilburg WAP, Smoktunowicz E (2021). Affirming basic psychological needs promotes mental well-being during the Covid-19 outbreak. Social Psychological and Personality Science.

[CR13] Chen B, Vansteenkiste M, Beyers W, Boone L, Deci EL, van der Kaap-Deeder J, Duriez B, Lens W, Matos L, Mouratidis A, Ryan RM, Sheldon KM, Soenens B, van Petegem S, Verstuyf J (2015). Basic psychological need satisfaction, need frustration, and need strength across four cultures. Motivation and Emotion.

[CR14] Cheon SH, Reeve J, Lee Y, Ntoumanis N, Gillet N, Kim BR, Song Y-G (2019). Expanding autonomy psychological need states from two (satisfaction, frustration) to three (dissatisfaction): A classroom-based intervention study. Journal of Educational Psychology.

[CR15] Cheon SH, Reeve J, Vansteenkiste M (2020). When teachers learn how to provide classroom structure in an autonomy-supportive way: Benefits to teachers and their students. Teaching and Teacher Education.

[CR16] Chirkov V, Ryan RM, Kim Y, Kaplan U (2003). Differentiating autonomy from individualism and independence: A self-determination theory perspective on internalization of cultural orientations and well-being. Journal of Personality and Social Psychology.

[CR17] Cohen S, Alper CM, Doyle WJ, Treanor JJ, Turner RB (2006). Positive emotional style predicts resistance to illness after experimental exposure to rhinovirus or influenza a virus. Psychosomatic Medicine.

[CR18] Costa S, Soenens B, Gugliandolo MC, Cuzzocrea F, Larcan R (2015). The mediating role of experiences of need satisfaction in associations between parental psychological control and internalizing problems: A study among Italian college students. Journal of Child and Family Studies.

[CR19] Creswell, J. W., & Plano Clark, V. L. (2018). *Designing and conducting mixed methods research*. Sage.

[CR20] Deci EL, Ryan RM (2008). Facilitating optimal motivation and psychological well-being across life’s domains. Canadian Psychology.

[CR21] Deci EL, Ryan RM (2011). Levels of analysis, regnant causes of behavior and well-being: The role of psychological needs. Psychological Inquiry.

[CR22] Fawaz M, Al Nakhal M, Itani M (2021). COVID-19 quarantine stressors and management among Lebanese students: A qualitative study. Current Psychology.

[CR23] Fetters MD, Curry LA, Creswell JW (2013). Achieving integration in mixed methods designs: Principles and practices. Health Services Research.

[CR24] Flora DB, Flake JK (2017). The purpose and practice of exploratory and confirmatory factor analysis in psychological research: Decisions for scale development and validation. Canadian Journal of Behavioural Science.

[CR25] Gläser-Zikuda M, Hagenauer G, Stephan M (2020). The potential of qualitative content analysis for empirical educational research. Forum: Qualitative Social Research.

[CR26] Haerens L, Aelterman N, Vansteenkiste M, Soenens B, van Petegem S (2015). Do perceived autonomy-supportive and controlling teaching relate to physical education students’ motivational experiences through unique pathways? Distinguishing between the bright and dark side of motivation. Psychology of Sport and Exercise.

[CR27] Hagenauer, G., & Raufelder, D. (2021). Einleitung [Introduction]. In G. Hagenauer & D. Raufelder (Eds.), *Soziale Eingebundenheit: Sozialbeziehungen im Fokus von Schule und Lehrer*innenbildung [Social Relatedness: Social relations in school and in teacher education]*. Waxmann.

[CR28] Heissel A, Pietrek A, Flunger B, Fydrich T, Rapp MA, Heinzel S, Vansteenkiste M (2018). The validation of the German basic psychological need satisfaction and frustration scale in the context of mental health. European Journal of Health Psychology.

[CR29] Iacobucci D, Saldanha N, Deng X (2007). A meditation on mediation: Evidence that structural equations models perform better than regressions. Journal of Consumer Psychology.

[CR30] Johnson, B., & Christensen, L. (2004). *Educational research: Quantitative, qualitative and mixed approaches* (2nd ed.). Pearson.

[CR31] Kanuka H, Heller B, Jugdev K (2008). The factor structure of teaching development needs for distance-delivered e-learning. International Journal for Academic Development.

[CR32] Klassen RM, Perry NE, Frenzel AC (2012). Teachers’ relatedness with students: An underemphasized component of teachers’ basic psychological needs. Journal of Educational Psychology.

[CR33] Kumar A, Nayar KR (2020). Covid 19 and its mental health consequences. Journal of Mental Health.

[CR34] Little RJA (1988). A test of Missing Completely at Random for multivariate data with missing values. Journal of the American Statistical Association.

[CR35] Lunenberg M, Korthagen F, Swennen A (2007). The teacher educator as role model. Teaching and Teacher Education.

[CR36] Martela F, Sheldon KM (2019). Clarifying the concept of well-being: Psychological need satisfaction as the common core connecting eudaimonic and subjective well-being. Review of General Psychology.

[CR37] Martinek D, Zumbach J, Carmignola M (2020). The impact of perceived autonomy support and autonomy orientation on orientations towards teaching and self-regulation at university. International Journal of Educational Research.

[CR38] Mayring, P. (2014). *Qualitative content analysis: Theoretical foundation, basic procedures and software solution*. https://nbn-resolving.org/urn:nbn:de:0168-ssoar-395173.

[CR39] Murphy MPA (2020). COVID-19 and emergency eLearning: Consequences of the securitization of higher education for post-pandemic pedagogy. Contemporary Security Policy.

[CR40] Paulhus DL, Robins RW, Trzesniewski KH, Tracy JL (2004). Two replicable suppressor situations in personality research. Multivariate Behavioral Research.

[CR41] Penninx BWJH, Deeg DJH, van Eijk JTM, Beekman ATF, Guralnik JM (2000). Changes in depression and physical decline in older adults: A longitudinal perspective. Journal of Affective Disorders.

[CR42] Pichl, E. (2020). Hochschulrecht und -governance im „COVID-19-Modus“ – ein erstes Crossover: [Law and governance of institutions of higher education in COVID-19-mode]. *Zfhr*, *19*(3), 75. 10.33196/zfhr202003007501.

[CR43] Piedmont, R. L. (2014). Beta weights. In A. C. Michalos (Ed.). Encyclopedia of quality of life and well-being research (pp. 381–381). Springer, Netherlands. 10.1007/978-94-007-0753-5_201.

[CR44] Polk DE, Cohen S, Doyle WJ, Skoner DP, Kirschbaum C (2005). State and trait affect as predictors of salivary cortisol in healthy adults. Psychoneuroendocrinology.

[CR45] R Core Team. (2017). R: A language and environment for statistical computing. Vienna, Austria. https://www.R-project.org/.

[CR46] Reeve, J. (2018). *Understanding motivation and emotion*. John Wiley & Sons.

[CR47] Rosseel, Y. (2012). lavaan: An R Package for structural equation modeling. *Journal of Statistical Software*, 48(2). 10.18637/jss.v048.i02.

[CR48] Ryan RM, Bernstein JH, Brown KW (2010). Weekends, work, and well-being: Psychological need satisfactions and day of the week effects on mood, vitality, and physical symptoms. Journal of Social and Clinical Psychology.

[CR49] Ryan RM, Deci EL (2000). Self-determination theory and the facilitation of intrinsic motivation, social development, and well-being. American Psychologist.

[CR50] Ryan RM, Deci EL (2001). On happiness and human potentials: A review of research on hedonic and eudaimonic well-being. Annual Review of Psychology.

[CR51] Ryan RM, Deci EL (2008). From ego depletion to vitality: Theory and findings concerning the facilitation of energy available to the self. Social and Personality Psychology Compass.

[CR52] Ryan, R. M., & Deci, E. L. (2011). A self-determination theory perspective on social, institutional, cultural, and economic supports for autonomy and their importance for well-being. In V. I. Chirkov, R. M. Ryan, & K. M. Sheldon (Eds.), *Human Autonomy in cross-cultural context* (pp. 45–64). Springer.

[CR53] Ryan, R. M., & Deci, E. L. (2017). *Self-determination theory: Basic psychological needs in motivation, development, and wellness*. The Guilford Press.

[CR54] Ryan RM, Frederick C (1997). On energy, personality, and health: Subjective vitality as a dynamic reflection of well-being. Journal of Personality.

[CR55] Ryan RM, Rigby CS, Przybylski A (2006). The motivational pull of video games: A Self-Determination Theory approach. Motivation and Emotion.

[CR56] Schreiber JB, Stage FK, King J, Nora A, Barlow EA (2006). Reporting structural equation modeling and confirmatory factor analysis results: A review. The Journal of Educational Research.

[CR57] Shieh G (2006). Suppression situations in multiple linear regression. Educational and Psychological Measurement.

[CR58] Smolders KCHJ, de Kort YAW, van den Berg SM (2013). Daytime light exposure and feelings of vitality: Results of a field study during regular weekdays. Journal of Environmental Psychology.

[CR59] VERBI Software. (2019). *MAXQDA 2020* [Computer software]. Berlin, Germany. maxqda.com.

[CR60] Uerz D, Volman M, Kral M (2018). Teacher educators’ competences in fostering student teachers’ proficiency in teaching and learning with technology: An overview of relevant research literature. Teaching and Teacher Education.

[CR61] Uribe SN, Vaughan M (2017). Facilitating student learning in distance education: A case study on the development and implementation of a multifaceted feedback system. Distance Education.

[CR62] van den Broeck A, Ferris DL, Chang C-H, Rosen CC (2016). A review of Self-Determination Theory’s basic psychological needs at work. Journal of Management.

[CR63] van den Broeck A, Vansteenkiste M, de Witte H, Lens W (2008). Explaining the relationships between job characteristics, burnout, and engagement: The role of basic psychological need satisfaction. Work & Stress.

[CR64] Vansteenkiste M, Ryan RM (2013). On psychological growth and vulnerability: Basic psychological need satisfaction and need frustration as a unifying principle. Journal of Psychotherapy Integration.

[CR65] Vansteenkiste M, Ryan RM, Soenens B (2020). Basic psychological need theory: Advancements, critical themes, and future directions. Motivation and Emotion.

[CR66] Vermote B, Waterschoot J, Morbée S, Van der Kaap-Deeder J, Schrooyen C, Soenens B, Ryan R, Vansteenkiste M (2021). Do psychological needs play a role in times of uncertainty? Associations with well-being during the COVID-19 crisis. Journal of Happiness Studies.

[CR67] Wang C, Hsu H-CK, Bonem EM, Moss JD, Yu S, Nelson DB, Levesque-Bristol C (2019). Need satisfaction and need dissatisfaction: A comparative study of online and face-to-face learning contexts. Computers in Human Behavior.

[CR68] Watson D, Tellegen A (1985). Toward a consensual structure of mood. Psychological Bulletin.

[CR69] Weinstein N, Ryan RM (2010). When helping helps: Autonomous motivation for prosocial behavior and its influence on well-being for the helper and recipient. Journal of Personality and Social Psychology.

[CR70] Zhao X, Lynch JG, Chen Q (2010). Reconsidering Baron and Kenny: Myths and truths about mediation analysis. Journal of Consumer Research.

